# Effect of flashlight guidance on manual ventilation performance in cardiopulmonary resuscitation: A randomized controlled simulation study

**DOI:** 10.1371/journal.pone.0198907

**Published:** 2018-06-13

**Authors:** Ji Hoon Kim, Jin Ho Beom, Je Sung You, Junho Cho, In Kyung Min, Hyun Soo Chung

**Affiliations:** 1 Department of Emergency Medicine, College of Medicine, Yonsei University, Seoul, Korea; 2 Department of Biostatistics Collaboration Unit, College of Medicine, Yonsei University, Seoul, Korea; University of Oklahoma, UNITED STATES

## Abstract

Several auditory-based feedback devices have been developed to improve the quality of ventilation performance during cardiopulmonary resuscitation (CPR), but their effectiveness has not been proven in actual CPR situations. In the present study, we investigated the effectiveness of visual flashlight guidance in maintaining high-quality ventilation performance. We conducted a simulation-based, randomized, parallel trial including 121 senior medical students. All participants were randomized to perform ventilation during 2 minutes of CPR with or without flashlight guidance. For each participant, we measured mean ventilation rate as a primary outcome and ventilation volume, inspiration velocity, and ventilation interval as secondary outcomes using a computerized device system. Mean ventilation rate did not significantly differ between flashlight guidance and control groups (P = 0.159), but participants in the flashlight guidance group exhibited significantly less variation in ventilation rate than participants in the control group (P<0.001). Ventilation interval was also more regular among participants in the flashlight guidance group. Our results demonstrate that flashlight guidance is effective in maintaining a constant ventilation rate and interval. If confirmed by further studies in clinical practice, flashlight guidance could be expected to improve the quality of ventilation performed during CPR.

## Introduction

Guidelines from the American Heart Association (AHA) recommend that providers deliver one breath every 6 seconds (10 breaths/minute) after placement of an advanced airway while performing continuous chest compressions during cardiopulmonary resuscitation (CPR) [1]. Previous studies support this recommendation, showing that excessive ventilation rate increases intrathoracic pressure, limiting venous return and coronary perfusion pressure and thus resulting in lower survival [2, 3]. Excessive ventilation rate also leads to cerebral vasoconstriction due to a decline in the partial pressure of carbon dioxide in the blood and decreases cerebral blood flow [4]. Clinical studies report that ventilation rates greater than of 10 breaths/minute are common during CPR in adults and children, even when performed by health care providers [5–10].

Many methods have been devised to precisely control the rate of ventilation during CPR with an advanced airway [11]. Thoracic impedance measurement requires that providers choose a ventilation rate and volume and presents real-time feedback [9, 12]. Another method of controlling ventilation rate during resuscitation is capnography. However, both of these methods underestimate the true ventilation rate due to interruptions from multiple sources, including chest compressions during resuscitation [13, 14]. Several studies demonstrate that the use of a metronome during CPR helps achieve an accurate ventilation rate after intubation [15, 16]. However, when CPR is performed in the clinical field, noise from providers’ voices for instruction and medical equipment for resuscitation can interfere with the guide sound from the metronome [17]. Thus, we hypothesized that a flashlight could effectively guide ventilation performance during CPR.

The aim of this simulation-based study was to investigate whether using a flashlight as a simple visual guidance device can increase ventilation rate accuracy when unexperienced providers perform ventilation during CPR.

## Materials and methods

### Study design and participants

This study was approved by the Yonsei University Institutional Review Board (approval number 4-2016-0696) and performed in accordance with the ethical standards of the Declaration of Helsinki. We performed a prospective, randomized, parallel trial using a mannequin for evaluating ventilation performance. Senior medical students participating in an emergency medicine clinical clerkship from March 2017 to August 2017 at Yonsei University College of Medicine were recruited voluntarily. Researchers explained the purpose of the study to all participants, and written informed consent was obtained. However, participants were blind to the variables collected. A total of 121 medical students were included in this study after excluding students who did not want to participate or had physical problems preventing performance of CPR, including ventilation.

### Study protocol

**Fig 1:**
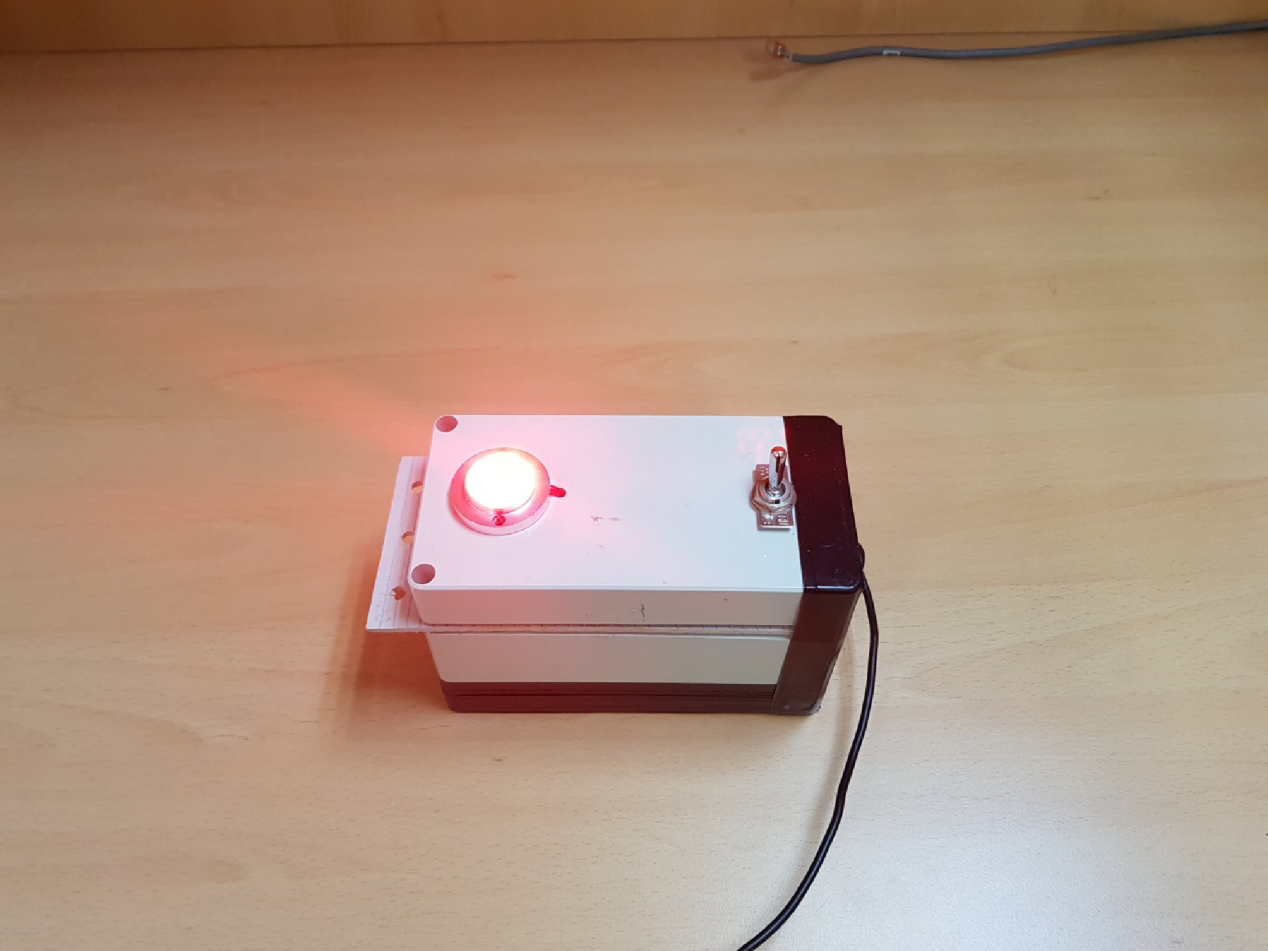
Image of the flashlight guidance device.

All participants completed 80 hours of regular college curriculum for 2 weeks. This curriculum included 3 hours of lecture, 6 hours of simulation sessions, and 10 hours of practical training in the clinical field for teaching advanced life support according to 2015 AHA guidelines. The intervention for the study was conducted on the last day of the clerkship. After informed consent, participants were randomized using a computer-generated random sequence into the flashlight guidance group or the control group. Before the intervention, all participants received 30 minutes of instruction on how to perform resuscitation involving ventilation support using a bag valve mask to intubated patients during CPR. To ensure that participants did not know that ventilation performance was the main outcome of the study, instructions concerned all aspects of CPR performance. For the flashlight guidance group, participants were instructed to perform ventilation synchronized with the rate of flashlight guidance. We manufactured the flashlight guide device for our study (Fig 1). The device was designed to turn on for 1 second every 6 seconds. The flashlight guide device was placed 5 m in front of the participant performing ventilation. For the control group, participants were instructed to achieve the ideal rate of ventilation without a flashlight. All participants were trained to supply 500 ml of ventilation capacity at a time according to AHA guidelines. After completion of instruction, participants performed ventilation in a simulated case of in-hospital CPR after advanced airway placement. Assessment was performed in groups of three participants performing CPR for 2 minutes in an independent simulation room. Two participants alternately performed chest compression, and a target participant was responsible for ventilation. All participants performed the simulation three times—once in a ventilation role and twice in a chest compression role. During the simulation, we played background noise using field recordings at a similar decibel as that in an actual hospital CPR situation.

### Data collection

Baseline characteristics of participants were collected before the intervention. We collected data on age, sex, body mass index (BMI), and experience with ventilation of actual intubated patients. A BT-CPEA® mannequin (17 kg; BT Inc., Wonju, Korea) capable of recording inspiration time and rate, ventilation volume and frequency per min, and chest compression rate in real time was used. Performance data were transmitted to and stored on a laptop computer. All participants were asked to judge the difficulty of performing the task after the simulation. Perceived difficulty was assessed using a 100-mm visual analog scale, with 0 being the easiest and 10 being the most difficult.

### Outcome measures

The primary outcome was mean ventilation rate. Secondary outcomes were mean ventilation volume, inspiration time, and ventilation interval.

### Statistical analysis

Sample size was calculated based on the primary outcome; ventilation rate was 22 breaths/minute without guidance in a study involving medical students [18]. We specified that an intervention producing a mean difference of 4 breaths/minute with a standard deviation difference of 2 would be considered clinically significant (P<0.05, statistical power = 80%). Therefore, the necessary sample size was determined to be 53, requiring a total of 118 participants considering a 10% dropout rate. Categorical data are presented as counts and percentages. Continuous data are presented as mean and standard deviation (SD) for normally distributed variables or as median and interquartile range (IQR) for non-normally distributed variables. To analyze differences between groups, we used Student’s t-tests or Mann-Whitney U tests for continuous variables and Chi-square tests or Fisher’s exact tests for categorical variables. The Brown-Forsythe test was used to analyze differences in group variance, as the test is robust against biases resulting from a failure to meet the normality assumption. P-values <0.05 were considered statistically significant. Data were analyzed using SAS, version 9.4 (SAS Institute Inc., Cary, NC, USA).

## Results

**Fig 2:**
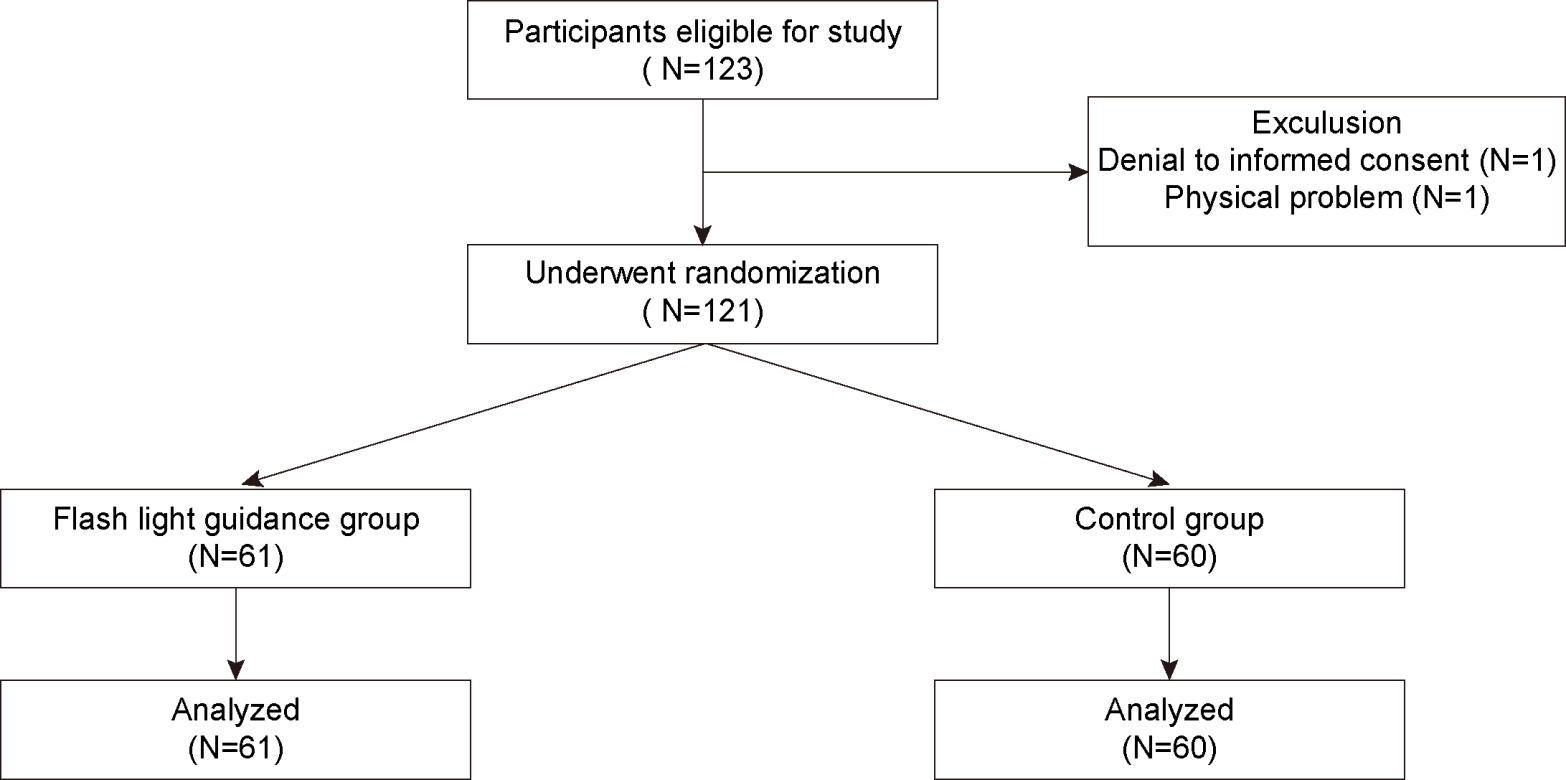
CONSORT participant flow chart.

Of the 123 eligible study participants, one student refused to enroll in the study and one was excluded due to upper extremity injury (Fig 2). Therefore, 61 participants were assigned to the flashlight guidance group, and 60 participants were assigned to the control group. There was no dropout after randomization. Participants in both groups showed similar baseline characteristics (Table 1).

**Table 1:** Participant characteristics.

		Flashlight group (n = 61)	Control group (n = 60)	Standardized difference
**Age (years)**		24.90 (2.05)	25.20 (2.13)	0.143
**Sex**	Female	21 (34.43%)	19 (31.67%)	0.059
	Male	40 (65.57%)	41 (68.33%)	
**BMI**		22.30 (2.40)	22.05 (2.91)	0.096
**Ventilation with mask bag**	Experienced	29 (47.54%)	23 (38.33%)	0.187
	Unexperienced	32 (52.46%)	37 (61.67%)	
**Actual CPR experience**	Experienced	28 (45.90%)	30 (50.00%)	0.082
	Unexperienced	33 (54.10%)	30 (50.00%)	
**Compression rate (min**^**-1**^**)**		116.18 (7.61)	116.48 (9.81)	0.035

Values are mean (SD) or number (proportion). CPR = cardiopulmonary resuscitation; BMI = body mass index.

**Fig 3:**
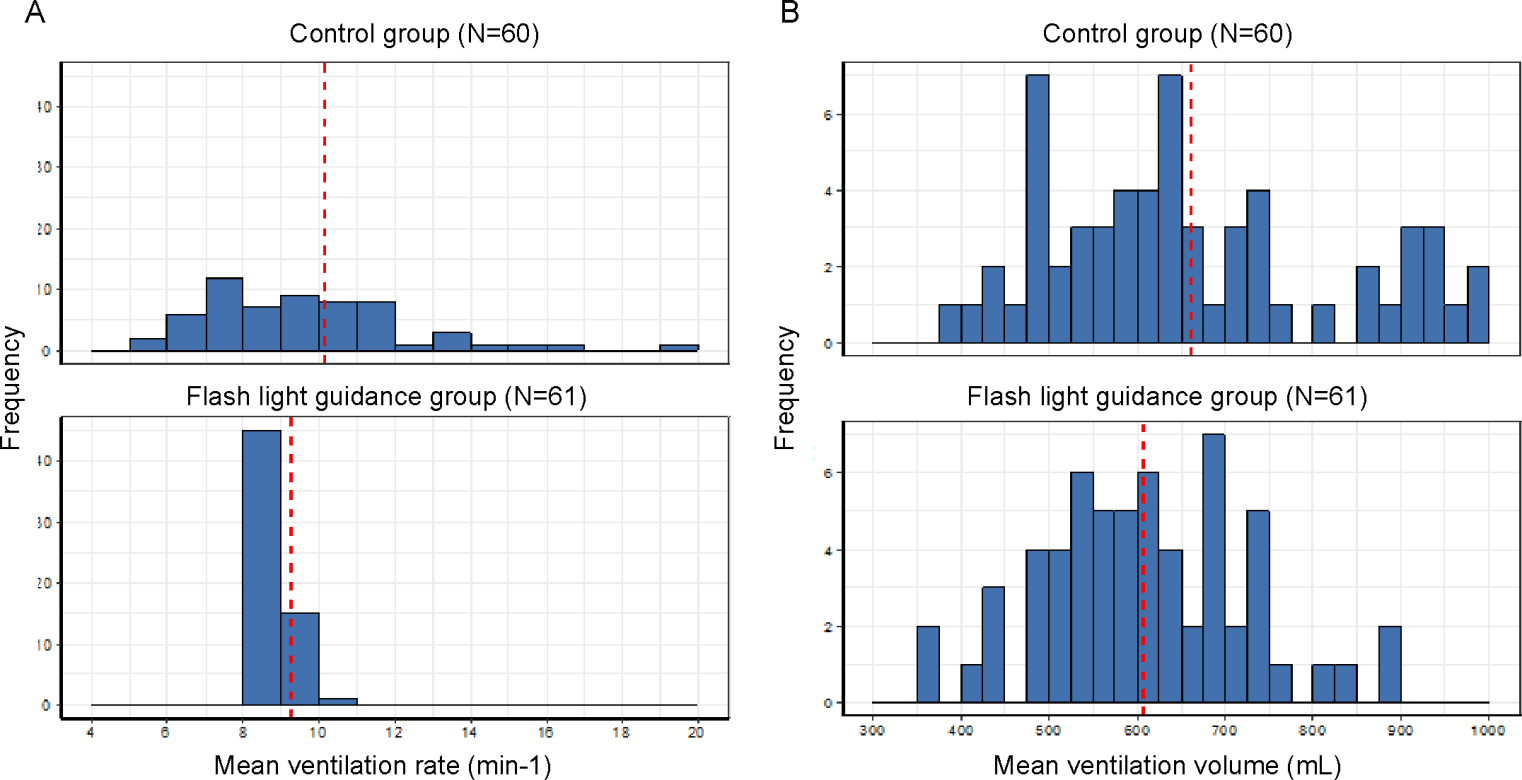
**Frequency histograms of mean ventilation rate (A) and ventilation volume (B) in the flashlight guidance and control groups.** The red dotted line indicates the mean value of individual datapoints.

During simulated CPR, mean ventilation rate was 9.00 (9.00–10.00) breaths/minute in the flashlight guidance group and 10.00 (8.00–12.00) breaths/minute in the control group (Table 2). Although there was no significant difference between groups (P = 0.159), the difference in variance between groups was significant (P<0.001). Mean ventilation volume was 605.00 (531.00–690.00) ml in the flashlight guidance group and 631.50 (530.75–748.50) ml in the control group. Again, although there was no significant difference between groups (P = 0.144), the difference in variance between groups was significant (P = 0.018). This is reflected by the smaller standard deviations for measures related to ventilation performance in the flashlight group (Fig 3). Mean inspiration velocities were 710.00 (636.00–822.00) and 524.50 (452.50–615.00) mL/s for the flashlight and control groups, respectively, which was a significant difference (P<0.001). Participants in the flashlight group reported significantly less difficulty in performing ventilation than participants in the control group (P<0.001).

**Table 2:** Ventilation performance during simulated CPR.

	Flashlight group (n = 61)	Control group (n = 60)	Mean difference P-value	Variance difference P-value
**Mean ventilation rate (min**^**-1**^**)**	9.00 (9.00–10.00)	10.00 (8.00–12.00)	0.159	<0.001
**Mean ventilation volume (mL)**	605.00 (531.00–690.00)	631.50 (530.75–748.50)	0.144	0.018
**Mean inspiration velocity (mL/s)**	710.00 (636.00–822.00)	524.50 (452.50–615.00)	<0.001	0.722
**Perceived difficulty**	2.00 (1.00–4.00)	6.00 (3.00–7.00)	<0.001	0.652

Values are median (IQR). Mean differences were analyzed using Mann Whitney U tests. Variance differences were analyzed using Brown-Forsythe’s tests.

The proportion of participants providing optimal ventilation volume was not significantly different between groups (P = 0.116). However, the proportion of participants providing optimal inspiratory duration was higher in the flashlight group than in the control group (P<0.001; Table 3).

**Table 3:** Proportion of participants providing optimal ventilation.

		Mean (SD)		Median (IQR)		
		Control group	Flashlight group	Control group	Flashlight group	P-value
		(n = 60)	(n = 61)	(n = 60)	(n = 61)	
**Ventilation volume (%)**	Optimal volume supply	35.74 (33.87)	44.56 (32.95)	28.17 (0.00–58.42)	45.00 (19.05–78.26)	0.116
	Excessive volume supply	51.12 (39.04)	41.00 (38.10)	57.54 (7.55–89.39)	40.00 (0.00–80.00)	0.141
	Insufficient volume supply	13.33 (23.33)	12.99 (21.50)	3.90 (0.00–13.12)	4.76 (0.00–15.00)	0.885
**Inspiration duration (%)**	Optimal inspiration duration	62.11 (38.09)	89.48 (17.03)	80.38 (22.66–95.39)	95.00 (90.00–100.00)	0.001
	Short inspiration duration	3.94 (13.74)	3.88 (6.83)	0.00 (0.00–0.83)	0.00 (0.00–5.00)	0.080
	Long inspiration duration	32.48 (36.08)	5.93 (16.58)	16.23 (0.00–62.31)	0.00 (0.00–0.00)	<0.001

Differences were analyzed using Mann Whitney U tests. Optimal volume supply was defined as ventilation volume was between 400 and 700 mL. Optimal inspiration duration was defined as inspiration duration was between 0.5 and 1.5 second.

**Fig 4:**
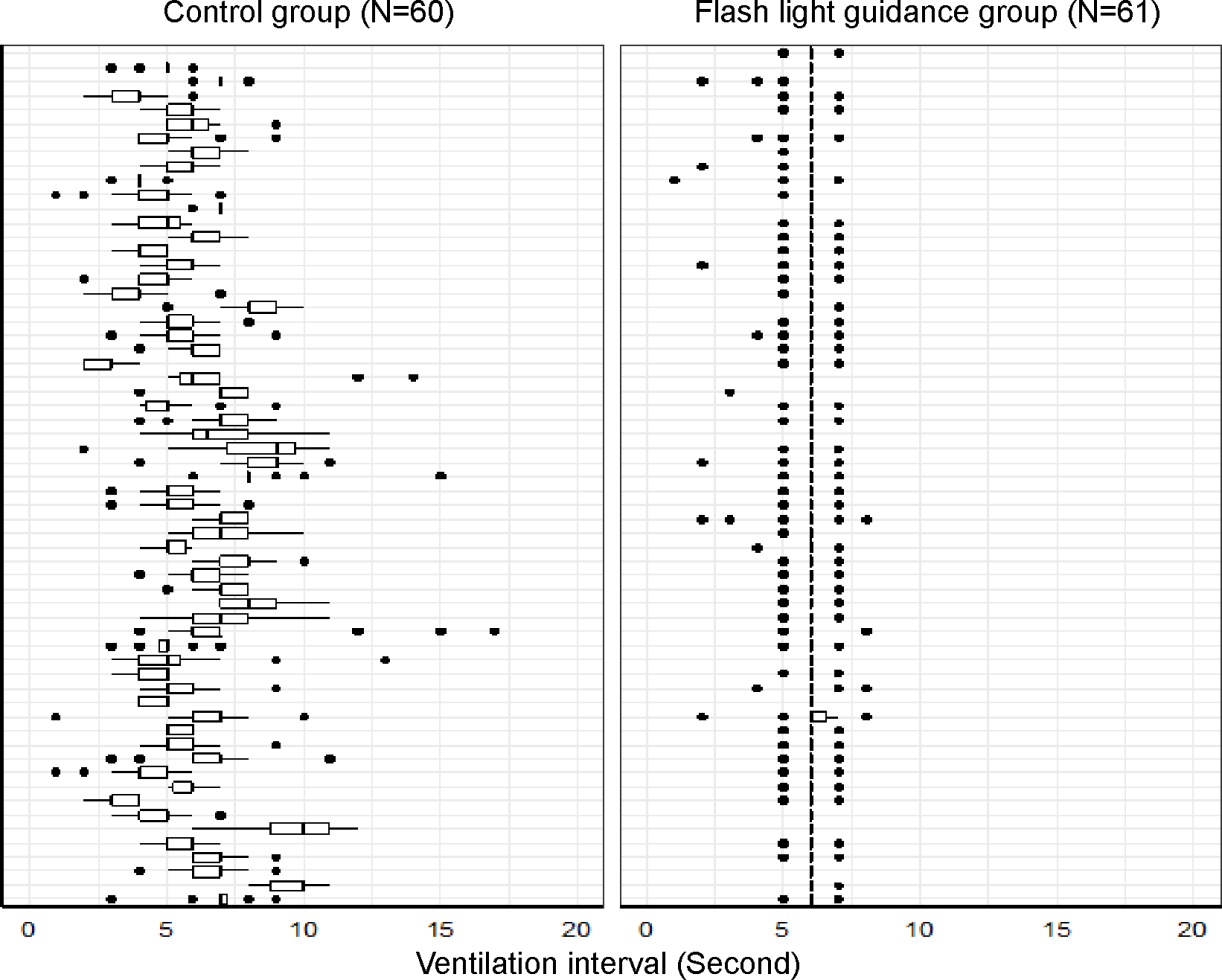
Boxplots representing ventilation interval during CPR in the flashlight guidance and control groups.

Fig 4 shows an overview of individual ventilation intervals during 2 min of CPR for participants in the flashlight and control groups.

## Discussion

We found that mean ventilation rate and volume were similar between the flashlight guidance and control groups, but flashlight guidance reduced variance in ventilation performance among participants.

The reason for the lack of difference in mean ventilation rate between groups appeared to be because the control group had similar proportions of participants performing ventilation faster or slower than AHA guidelines. Previous studies show that many providers regardless of experience do not maintain an accurate ventilation rate, especially in clinical settings, frequently resulting in excessive ventilation rates [5, 7, 11, 19]. Zhou *et al*. reported that the ventilation rate of medical students was 10 breaths/minute higher than that of physicians in actual CPR situations [18]. However, the ventilation rate of providers without intervention was not markedly higher in our simulation-based study than in actual CPR situations [20–22]. It is assumed that the urgency of actual CPR situations can disrupt the attention of the provider responsible for ventilation. In our study, participants were likely to have focused more on following AHA guidelines than usual because their ventilation performance was evaluated at the end of an emergency medicine clinical clerkship that involved training in CPR guidelines. Nevertheless, we found that the variance in ventilation rate significantly differed between groups, indicating that ventilation rate was not constant across individual participants in the control group. In other words, we found that flashlight guidance can help maintain a constant rate of ventilation that should be performed during CPR regardless of the individual performing the ventilation.

Our study confirmed that a higher proportion of participants in the flashlight guidance group provided an appropriate duration of ventilation, as they were constantly led to provide ventilation only when the guiding device was on. This resulted in a difference not only in mean inspiratory velocity but also the variance of the ventilation volume supplied by participants. In other words, the flashlight helped precisely control the inspiration time, thereby reducing differences in ventilation volume among participants. However, our study also demonstrated that controlling inspiratory duration does not guarantee adequate ventilation volume.

We confirmed that ventilation providers using flashlight guidance in a simulated CPR situation maintained a regular ventilation interval during 2 minutes of resuscitation compared with providers without guidance. Generally, a provider’s performance declines rapidly over time during CPR [23], which warrants the use of a guidance device. CPR is a process in which multiple providers play simultaneous roles, so that each provider should perform their specific role while also monitoring the overall flow [1]. However, as CPR time increases, providers become more fatigued, making it difficult to maintain concentration. In addition, unexpected situations may occur during CPR that prevent the ventilation provider from focusing on adequate ventilation. Therefore, flashlight guidance could help providers perform ventilation constantly regardless of other situations occurring during CPR.

Actual CPR is often performed in situations with substantial background noise that can saturate an auditory stimulus [17], thus leading to poorer CPR performance and increased risk of clinical error [24]. Although several auditory-based feedback devices have been introduced, they have not been proven effective in actual CPR situations [15, 25, 26]. Visual guidance could be more effective than auditory guidance in helping maintain a constant interval and rate of ventilation in a noisy CPR situation. We constructed the simulation using field recordings of an actual CPR situation as background noise, but this did not interrupt the maintenance of a regular ventilation rate and interval for participants under flashlight guidance.

The present study suggests a new concept for ventilation guidance during CPR. So far, previous studies have examined the effect of real-time feedback devices to improve ventilation quality during CPR. These studies have mainly used thoracic impedance or capnography as real-time feedback devices, although the information from these devices can be inaccurate because chest compression artifacts during CPR prevent real ventilation from being precisely detected [12–14, 27, 28]. Performance guiding using a physiological index obtained from patients is also unreliable because patients are subjected to a variety of physical stimuli during actual resuscitation. Therefore, guidance that the provider can actively refer to may be more effective during CPR, and visual-based devices may be more useful than audio-based devices like a metronome. We found that flashlight guidance can reduce individual differences among providers, thus demonstrating that standardized guidelines for ventilation during CPR can be followed by anyone. Also, flashlight guidance may be more useful in different settings including in resource-limited environment as well as developed nations where could be easily available next-generation simulators with automatic feedback technology. Lastly, as we evaluated medical students who did not yet have actual clinical experience, this study population was suitable for assessing educational effects on unskillful providers.

Our study has several limitations. As our study is simulation-based, it is necessary to validate the usefulness of flashlight guidance in clinical practice. In particular, CPR performed in a pre-hospital setting may involve factors that prevent detection of a visual signal [17]. Also, we found that flashlight guidance may not help achieve accurate ventilation volume during CPR. However, this guidance is designed to maintain only ventilation rate and interval; therefore, its combination with capacity-modulating intervention could help improve ventilation quality during CPR. In addition, participants were all medical students who were unskilled in ventilation; therefore, the efficacy of flashlight guidance may be different for skilled providers. Finally, as the present study was not masked, it is possible that participants suspected its purpose, leading participants in the flashlight guidance group to concentrate more on their ventilation performance quality.

## Conclusions

We found that guiding ventilation performance using a flashlight can help keep ventilation rate and interval constant and achieve accurate inspiration duration regardless of who is providing the ventilation. If further studies confirm this improvement in performance in clinical practice, this flashlight guidance could be expected to improve the quality of ventilation performed during CPR.

## Supporting information

S1 File Study data. (XLSX)
